# Review: An integrated graphical tool for analysing impacts and services provided by livestock farming

**DOI:** 10.1017/S1751731119000351

**Published:** 2019-03-04

**Authors:** J. Ryschawy, B. Dumont, O. Therond, C. Donnars, J. Hendrickson, M. Benoit, M. Duru

**Affiliations:** 1 UMR AGIR, Université de Toulouse, INRA, 31326 Castanet-Tolosan, France; 2 Université Clermont Auvergne, INRA, VetAgro Sup, UMR Herbivores, 63122 Saint-Genès-Champanelle, France; 3 Université de Lorraine, INRA, LAE, F-68000 Colmar, France; 4 DEPE, INRA, 75338 Paris, France; 5 USDA-ARS, Northern Great Plains Research Laboratory, P.O. Box 459, Mandan, ND 58554-0459, USA

**Keywords:** ecosystem services, trade-off, sustainability, agroecology, socioecological approach

## Abstract

Livestock farming is criticized for negatively impacting the environment, concerns about animal welfare and the impact of excessive meat consumption on human health. However, livestock farming provides other underappreciated and poorly communicated benefits to society in terms of employment, product quality, cultural landscapes and carbon storage by grasslands. Few attempts have been made so far to simultaneously consider the services and impacts provided by livestock production. Here, we propose an integrated graphical tool, called the ‘barn’ to explicitly summarize the synergies and trade-offs between services and impacts provided by livestock farming. It illustrates livestock farming interacting with its physical, economic and social environment along five interfaces: (i) Markets, (ii) Work and employment, (iii) Inputs, (iv) Environment and climate, (v) Social and cultural factors. This graphical tool was then applied by comparing two contrasting livestock production areas (high livestock density *v.* grassland-based), and the dominant *v.* a niche system within a crop-livestock area. We showed the barn could be used for cross-comparisons of services and impacts across livestock production areas, and for multi-level analysis of services and impacts of livestock farming within a given area. The barn graphically summarizes the ecological and socio-economic aspects of livestock farming by explicitly representing multiple services and impacts of different systems in a simple yet informative way. Information for the five interfaces relies on available quantitative assessments from the literature or data sets, and on expert-knowledge for more qualitative factors, such as social and cultural ones. The ‘barn’ can also inform local stakeholders or policy-makers about potential opportunities and threats to the future of livestock farming in specific production areas. It has already been used as a pedagogical tool for teaching the diversity of services and impacts of livestock systems across Europe and is currently developed as a serious game for encouraging knowledge exchange and sharing different viewpoints between stakeholders.

## Implications

Livestock farming can provide a wide range of economic, environmental, cultural and social services at local, regional and global levels, many of which have been little quantified and poorly shared with consumers, stakeholders and policy-makers. We propose an integrated graphical approach that summarizes the ecological and socio-economic aspects of livestock farming by explicitly representing the whole range of services and impacts of different systems. This graphical tool is a powerful means to highlight the diversity of livestock farming, and to share knowledge and viewpoints in search of more sustainable options for livestock farming.

## Introduction

The sustainability of livestock systems is widely debated because of their negative impact on the environment, water, soil and air quality, biodiversity, and greenhouse gas emissions, and concerns about animal welfare and excessive meat consumption on human health. Environmental impacts have been emphasized by the Food and Agricultural Organization (FAO, 2006) report ‘Livestock’s long shadow’, and have been confirmed and clarified by the scientific literature (De Vries and de Boer, 2010; Lassaletta *et al*., 2014; Westhoek *et al*., 2015; Chaudhary and Kastner, 2016; Röös *et al*., 2016). However, the current evaluations have generally focussed on the negative impacts of livestock farming, with the exception of reviews and meta-analyses that stress the positive effects of grassland-based livestock farming (Scohier and Dumont, 2012; Rodríguez-Ortega *et al*., 2014) and integrated crop-livestock systems (Aguilar *et al*., 2015; Lemaire *et al*., 2015) with the subsequent benefits to ecosystem services for farmers and society. However, the multiple services provided by livestock farming systems have been rarely considered simultaneously. This applies to both the coexistence and the interactions between the services and impacts of different systems (Ryschawy *et al*., 2017a; Dumont *et al*., 2018b). Livestock effects are usually examined either by discipline (physiology, animal nutrition, etc.), dimension (environment, economy and social) or by organizational level (animal, farming system or production area), making it difficult to balance the multiple effects of different livestock production systems. One recent development does consider a broad range of indicators by analysing a ‘bundle of services’, that is, a set of ecosystem services that appear together repeatedly across sites and through time (Raudsepp-Hearne *et al*., 2010). Bundles of services (*sensu lato*, i.e. wider than ecosystem services) can be used to assess livestock system sustainability by simultaneously accounting for the ecological and socio-economic aspects of livestock farming across production areas. Social services include territorial vitality (e.g. employment in farms, in the agrofood industry, contribution to social cohesion) and cultural heritage (e.g. gastronomy, recreational landscape, emblematic breeds) (Plieninger *et al*., 2013; Beudou *et al*., 2017; Ryschawy *et al*., 2017a).

To our knowledge, there is no operational integrated tool that provides an assessment of livestock farming at the system and/or regional level, while also considering the different dimensions of livestock sustainability, and explicitly includes both impacts and services. Here, we developed a tool to analyse and represent the multiple attributes of different systems across a range of livestock production areas in a simple yet informative way. First, we review the available literature to evaluate the services and impacts provided by livestock farming. Then we show how this knowledge was used to develop a graphical tool that simultaneously accounts for the impacts and services provided by livestock farming; we apply this tool to the Tarn-Aveyron Basin, which is a region with a balance between livestock production and cash crops. Third, we apply this tool to two other contrasting livestock production areas, and to a niche system within the Tarn-Aveyron Basin. By doing this, we show the barn could be used to compare services and impacts across livestock production areas, and also for multi-level analysis of services and impacts of livestock farming within a given area. We conclude by discussing the strengths and weaknesses of this graphical tool.

## How to represent the diversity of services and impacts of livestock systems?

When impacts and services of livestock production are analysed and quantified the focus is often on relatively few services or impacts (Tancoigne *et al*., 2014) and the trade-offs between production and environmental dimensions (De Vries and de Boer, 2010; Scohier and Dumont, 2012; Sabatier *et al*., 2015). This contrasts with analysing the ‘bundle of services’ provided by livestock farming (Raudsepp-Hearne *et al*., 2010). Trade-offs between production and socio-cultural dimensions have been largely overlooked due to methodological challenges (Dolman *et al*., 2014; Beudou *et al*., 2017). Cultural services are difficult to quantify because they do not represent purely biophysical phenomena and are given different values by stakeholders. In addition, the emphasis on analysing impacts at field or farm levels has resulted in a lack of information and methods for use at higher scales. Table 1 provides an overview of the primary current methods for assessing the impacts and services of livestock farming, and their limits. Three main limits are discussed in the following sections: (i) a segregation between the evaluation of the negative impacts from agriculture and multi-functionality or ecosystem services frameworks, (ii) an emphasis on agro-environmental impacts over the social aspects and (iii) focussing at the farm level despite recent and promising development of social-ecological approaches at the regional level.

**Table 1:** Main methods developed for assessing the impacts and services of livestock systems ranging from farm to regional and food chain levels

Methods	Aspects of sustainability covered	Organizational level	Main limits	References
LCA, nutrient flows, ecobalances and footprint approach	*Environment*: resources and energy flows, biogeochemical cycles, water, soil and air pollutions, greenhouse gas emissions, wastes (biodiversity under development) *Economic*: employment (under development) *Cultural and social*: animal health and welfare	Product, farm, food chain, geographical region	Only quantifies impacts, not services and does not work with social impacts Does not explicitly show the synergies or trade-offs between dimensions and scales since the indicators are aggregated Sensitivity to functional unit (e.g. per unit area or per unit product)	van der Werf and Petit (2002) De Vries and de Boer (2010) Lassaletta *et al*. (2014)
Cost–benefits approach	*Environment*: resources and energy flows *Economic*: markets and food supply, employment, policies *Cultural and social*: animal health and welfare	Food-chain, region	Sensitivity to functional unit (e.g. performance per unit area or per unit product) Limited by data availability in large national databases	Mc Inerney *et al*. (1992) Yrjölä and Kola (2008)
Social and cultural qualitative analysis	*Environment*: not taken into account *Economic*: markets and food supply, policies, agro-tourism *Cultural and social*: cultural values, rural vitality	Farm, region	Often not quantified Time-consuming Hard to scale-up or transpose to another context Highly influenced by the interviewer’s point of view	Lamarque *et al*. (2011) Beudou *et al*. (2017)
Social-ecological approach, territorial metabolism	*Environment*: resources and energy flows, biodiversity, land-use/landscape structure, biogeochemical cycles, greenhouse gas emissions *Economic*: markets and food supply, policies *Cultural and social*: food safety, rural vitality	Region	Poorly deals with the farm level and technical approaches As with most case-study approaches, it is highly related to the regional context	Billen *et al*. (2014) Bonaudo *et al*. (2014) McGinnis and Ostrom (2014) Sabate *et al*. (2016)
Multi-functionality, multicriteria and ecosystem services approach	*Environment*: resources and energy flows, biodiversity, land-use/landscape structure, biogeochemical cycles, carbon storage, greenhouse gas emissions, water, soil and air pollutions *Economic*: markets and food supply, policies *Cultural and social*: food safety, rural vitality	Plot, landscape, region	Poorly deals with farm level and technical approaches Difficulty to integrate social aspects, due to limited data availability in large databases Often does not consider negative impacts explicitly, e.g., in bundle of services or multi-functionality approaches	MEA (2005) Zhang *et al*. (2007) Lescourret *et al*. (2015) Ryschawy *et al*. (2017a)
Scenario modelling and mapping	*Environment*: resources and energy flows, biological processes, water, soil and air pollutions, climate change, land-use/landscape *Economic*: markets and food supply, policies *Cultural and social*: food and protein supply, food security	Farm, region, country, EU, world	Lack of available quantitative indicators in national or global databases Models do not capture the social mechanisms behind transition (e.g. consumer preferences) Usually does not include social aspects Does not account for the diversity of livestock production areas	Westhoek *et al*. (2015) Chaudhary and Kastner (2016) Röös *et al*. (2016) Dumont *et al*. (2018b)

Information is given on the aspects of sustainability and domains covered, level of organization considered and main limits of such methods. Illustration of the methods are provided through selected references. LCA=Life Cycle Assessment.

### A segregation between the evaluation of the impacts and services

There has been an emphasis in the scientific literature on the negative environmental impacts of livestock systems (FAO, 2006; Westhoek *et al*., 2015). The impact of livestock production on the environment has been largely focussed on nutrient and material flow modelling, using either environmental impact assessment tools such as Life Cycle Assessment (LCA), which accounts for all the resources, flows and pollutant emissions associated with the life stages of a product (van der Werf and Petit, 2002; De Vries and de Boer, 2010), or stock and flow analysis (Fernandez-Mena *et al*., 2016). Conversely, the multi-functionality and ecosystem services frameworks explore a wide range of potential functions and benefits of livestock farming systems, beyond the production of animal proteins. Evaluations of farming systems include carbon storage (Smith, 2014) and flood control by grasslands, landscape aesthetic value, recreation and tourism potential, etc. This approach thus overcomes the ‘narrow’ focus of the domain- and level-based approaches. Ryschawy *et al*. (2017a) extended this approach to include territorial vitality (employment in farms, in R&D and the agrofood industry, stability of employment) and cultural identity (gastronomy, maintenance of hedges, emblematic breeds). However, this type of study is limited in that it does not explicitly account for the negative impacts of livestock production. As an example, when the negative impacts of livestock on water quality was evaluated, low values but not negative values were given for drinking water availability (Turner *et al*., 2014) or water quality (Ryschawy *et al*., 2017a). The negative impacts of livestock farming on water quality were thus only accounted for indirectly. However, both the negative environmental impacts and positive ecosystem services need to be simultaneously quantified when dealing with the biophysical effects of farming systems on the environment (Barot *et al*., 2017).

### An emphasis on agro-environmental impacts at the detriment of social aspects

Recent research has focused on developing quantitative agro-environmental indicators (Makowski *et al*., 2009; Dolman *et al*., 2014; Zehetmeier *et al*., 2014) by conducting experiments at plot and farm levels or utilizing national databases. At the same time, economists have concentrated on quantifying market-based flows, especially with cost–benefit approaches, and often only consider the economic dimensions and negative aspects of livestock farming (Mc Inerney *et al*., 1992; Pearce, 2003; Yrjölä and Kola, 2008). This focus on either agro-environmental or economic assessments has resulted in (i) a disconnect between the environmental, economic and social dimensions in the literature (Dumont *et al*. 2018b), and (ii) multicriteria evaluations of livestock farming that neglect their social dimensions (Oteros-Rozas *et al*., 2012; Rodríguez-Ortega *et al*., 2014).

However, recently there have been attempts to develop proxies for social indicators by using information available in databases, such as the number of working hours or the accident of farm workers per year (Röös *et al*., 2016) or the number of direct and indirect jobs created by the livestock sector (Ryschawy *et al*., 2017a). Beudou *et al*. (2017) developed a qualitative approach for evaluating social indicators of livestock systems in two contrasting livestock production areas. Their research revealed that social aspects of livestock production, including the territorial vitality and cultural services can greatly increase local acceptability of livestock farming and may potentially prevent the transition of livestock farming to less industrial systems. Although these works represent progress in better accounting for the social dimensions of livestock farming, they are still limited. There is a lack of indicators and it is difficult to account for shifts in consumer preferences and other social mechanisms that are behind transition to different food systems (Dumont *et al*., 2018b). The common technique of using interviews is also time-consuming, cannot be easily transposed to other contexts and the outputs cannot be easily quantified.

### A focus at farm level in spite of the development of social-ecological approaches

Evaluation of livestock systems is still focussed at the field/herd or farm level with less emphasis on the farm environment or multiple stakeholder preferences. Modelling approaches have successfully predicted the environmental impacts of livestock systems at the landscape level by using proxies of landscape heterogeneity that are assumed to benefit biodiversity (Sabatier *et al*., 2015), but these approaches do not consider any social aspects. More recently, the ‘social-ecological system’ framework has led to a better understanding of the complexity of the human–nature interactions on a range of simultaneously assessed environmental, social and economic dimensions (McGinnis and Ostrom, 2014). While initially developed to deal with natural resource management issues, this approach has been applied to agriculture and food systems, since it could include the physical space dedicated to production (including resources, infrastructure, markets, institutions) and people involved (Cabell and Oelofse, 2012; Lescourret *et al*., 2015; Vallejo-Rojas *et al*., 2015). The social-ecological system approaches place the stakeholders in the centre of the agriculture/food system, but they are less effective at considering the biotechnical aspects of farming systems and the interaction with humans and downstream channels (Duru *et al*., 2015; Marshall, 2015; Touzard *et al*., 2015). Although highly integrative, these approaches lack the ability to consider product quality, the impact of globalized markets and public policies, and the off-farm impacts embodied in international trade of livestock feed (Chaudhary and Kastner, 2016). Territorial metabolism studies, or more recently the concept of environmental nutrition (Sabate *et al*., 2016) connect natural processes with the social and technical characteristics of an area (Bonaudo *et al*., 2014). However, such approaches are rarely used for evaluating livestock production areas.

This short review of existing methodologies for assessing the services and impacts of livestock systems highlights the inadequacy of current approaches in simultaneously accounting for the whole range of economic, environmental and social dimensions of livestock faming. Therefore, we developed an integrated and operational tool that graphically summarizes the ecological and socio-economic aspects of livestock farming by explicitly representing the multiple services and impacts of different systems in a simple yet informative way.

## The ‘barn’: a graphical tool approach to analyse impacts and services provided by livestock farming: application to the Tarn-Aveyron Basin

### Objective and specifications of the tool

Drawing up a balance sheet with the positive and negative impacts of livestock farming systems is challenging given the weaknesses of existing frameworks to consider the multiplicity of services and impacts of livestock systems, their variability across livestock production areas and farming systems, and the uncertainty of some assessments. We thus developed a graphical tool that can represent the services and impacts provided by livestock systems, and be adapted from the farm to the regional level.

Such a tool would seek to:

∙ Provide an operational representation of the livestock system as a social-ecological system. In line with Marshall (2015) and Sabate *et al*. (2016) such a tool should consider the production, processing and consumption of livestock products, public perception of farming and food systems, and explicitly characterize the environmental and socioeconomic context of any livestock farming system.
∙ Provide the ability to simultaneously consider the environmental, economic and social impacts and services in the same graphic without minimizing any of these categories regardless of information or indicator availability. It should integrate quantitative indicators when available but also consider qualitative data and stakeholder knowledge (Bammer, 2005; Beudou *et al*., 2017).
∙ Account for all material flows required for and generated by livestock production, and for local and off-farm biophysical impacts on the environment (Billen *et al*., 2014; Fernandez-Mena *et al*., 2016). This is important for accounting for supply chains that develop across territories, which need to include off-farm impacts of livestock farming including the production of animal feed (Chaudhary and Kastner, 2016).

### Graphical representation of a crop-livestock region

**Figure 1:**
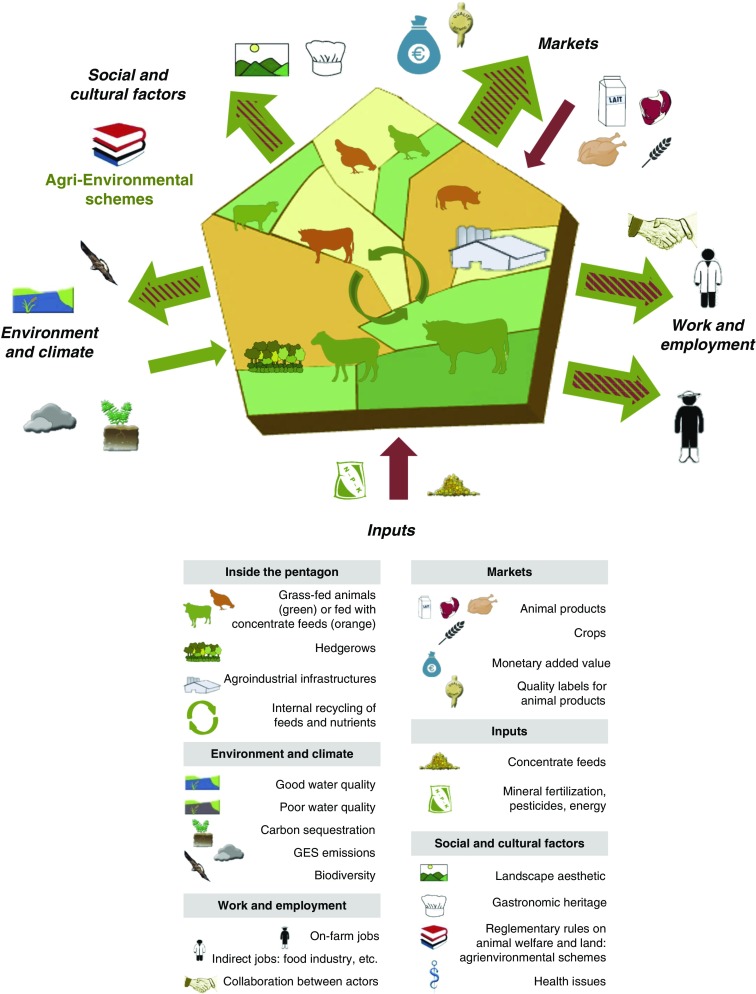
Conceptual framework applied to a crop-livestock region, the Tarn-Aveyron Basin (Moraine *et al*., 2016; Ryschawy *et al*., 2017b). The pentagon is spatially representing the livestock system considered. The diversity and proportion of land use is represented with two shades of green for permanent and temporary grasslands, and two shades of yellow to account for the diversity of crop rotations. Natural infrastructures (hedges, forests, etc.) and the agro-industrial buildings related to livestock production are also graphically represented. Balances between livestock production are symbolized by size of animal species pictograms (dairy and beef cattle, pigs, poultry, sheep). Grass-fed animals are green and livestock fed concentrates including maize silage and protein-rich feed cakes are represented by orange. Orange animals are more likely to be confined which is typical for intensive pig and poultry systems. The magnitude of impacts or services are represented by the size of outward-pointing arrows, and by their colour. Input services benefiting grassland-based systems are represented by a green inward-facing arrow on the *Environment and climate* interface. More globally arrow colours indicate positive (green), negative (red) or mixed (hatched with the dominant effect indicated by the colour of the outside border) effects.

Within these specifications, we developed a graphical tool that provides an integrated representation of a livestock farming system, seen as a social-ecological system. It is centred around a pentagon which represents the spatial boundaries of the system, either a farm, a livestock farming system or a livestock production area, such as in the example of the Tarn-Aveyron Basin (Ryschawy *et al*., 2017b), which is a region with a balance between livestock production (mainly ruminants and some poultry) and cash crops (Figure 1; Table 2). Because of its pentagonal appearance, we call our tool the ‘barn’. It consists of a multi-level graphical representation that can be used at different scales (farm, farming system or region) defined as county, watershed, farm-network or at the Nomenclature of Territorial Units for Statistics 3 level (NUTS3). Pentagon limits do not need to be linked to a specific administrative area, which allows further flexibility in the representation. Within the pentagon, two shades of green account for permanent and temporary grasslands, and two shades of yellow for the diversity of crop rotations. Natural and agro-industrial infrastructures are the main characteristics of livestock production areas in terms of nature and balance of livestock products and they are represented with intuitive pictograms (Figure 1). Grass-fed animals are in green, those fed concentrate feeds, including maize silage, are in orange. We consider not only interactions within the system (e.g. green circular arrows in Figure 1), but also interactions of the system along five interfaces: (i) Markets, (ii) Work and employment, (iii) Inputs, (iv) Environment and climate, (v) Social and cultural factors. Outward-pointing and inward-facing arrows around the pentagon allow visualizing the relative importance of variables represented along these five interfaces. The nature of the interaction is given by arrow colours, according to whether these effects are positive (green arrow), negative (red) or mixed (hatched).

**Table 2:** Table providing a synthesis of the services and impacts in a crop-livestock region: the Tarn-Aveyron Basin case-study

Interfaces	Positive effects – services	Negative effects – impacts
Inputs	Local production of livestock feed (Magne *et al*., 2016) is represented by green circular arrows within the barn	Off-farm impacts linked to soya bean imports (Magne *et al*., 2016) Mineral fertilization of crops; water used for maize (Murgue *et al*., 2015)
Labour and employment	Direct employment of farmers in many little farms (Ryschawy *et al*., 2017a) Indirect employment in local transformation of livestock products, cooperatives, etc. (Ryschawy *et al*., 2017a)	Low remuneration of work in farms and local supply chains
Markets	Lots of quality-labelled products and direct sale (Beudou *et al*., 2017)	Little added-value for young beef (Moraine *et al*., 2016) Fluctuating prices of crops on global market (Ryschawy *et al*., 2019)
Social and cultural factors	High nutritional quality of grass-fed ruminant products (Duru *et al*., 2017) Aesthetic of mosaic landscapes and gastronomy heritage (Beudou *et al*., 2017)	Conflict on the use of water, largely due to the high quantities required for maize silage (Murgue *et al*., 2015)
Environment and climate	High level of biodiversity in permanent grasslands and agroecological infrastructures (Moraine *et al*., 2016) Carbon storage in grasslands Water quality preservation	High level of CH_4_/ kg of product (Duru *et al*., 2017) Sensivity of grassland systems to climate, and of crops to pests (Magne *et al*., 2016)

### The barn represents how the system interacts along five interfaces

**Figure 2:**
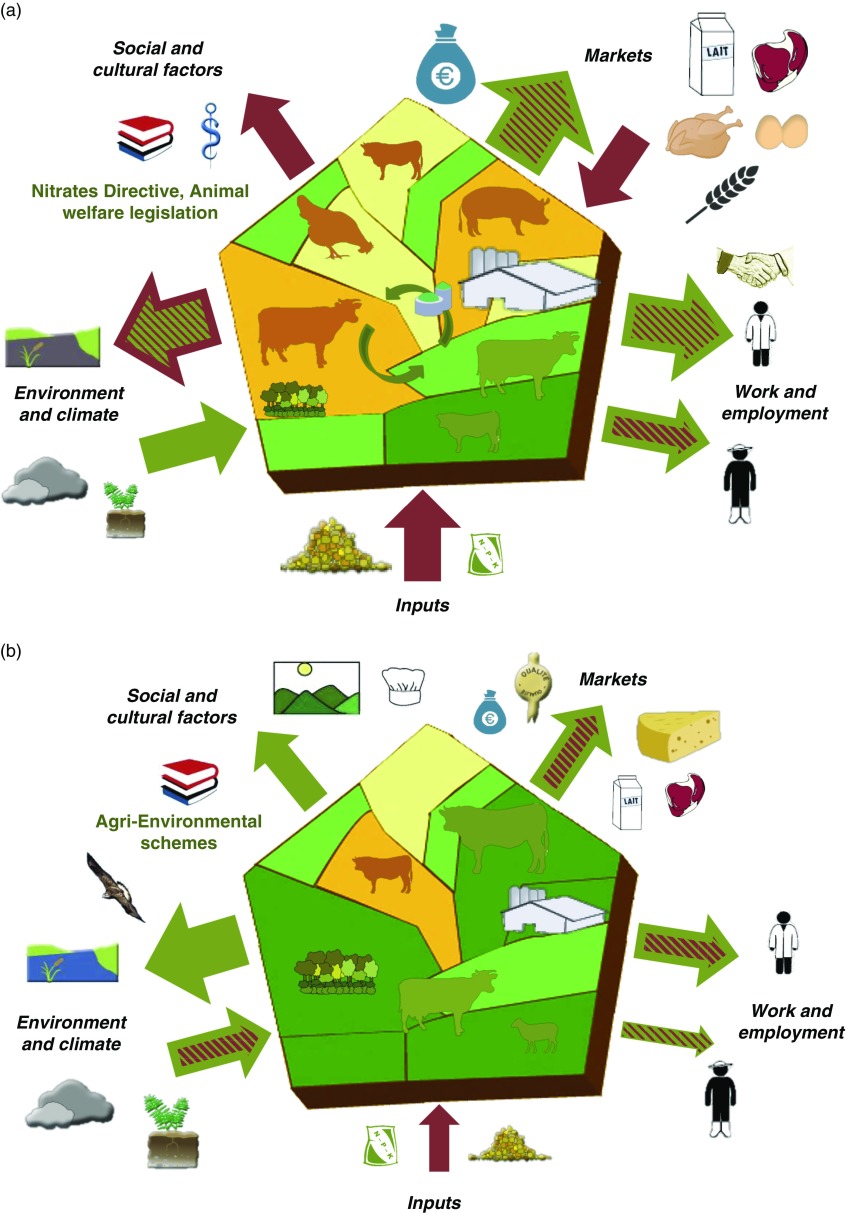
Contrasting examples of trade-offs and synergies between the sides of the ‘barn’. Brittany (a) is an example of high livestock density regions with a trade-off between high production levels and environmental and cultural impacts (Dourmad *et al*., 2017). Massif central (b) is an example of grassland-based regions with lower production levels but more synergies with environmental and cultural services (Duru *et al*., 2017; Vollet *et al*., 2017). See Figure 1 for pictogram meaning.
  As our graphical tool is explicitly derived from a social-ecological approach, a large emphasis was placed on the role of stakeholders. Following Marshall (2015), we separated stakeholders involved in the livestock sector into either on-farm or from indirect jobs, or citizens and consumers whose values and ethical considerations are accounted for on the social and cultural interface.
∙ Work and employment. This interface focuses on on-farm and indirect jobs (represented by two distinct pictograms), working conditions (modulating arrow colour) and organization of supply chains with a specific pictogram accounting for collaboration between actors. How livestock contributes to local employment, employee status (including stability of employment) and working conditions varies across livestock sectors (Hostiou and Fagon, 2012; Röös *et al*., 2016). Employment is a major opportunity in high animal densities regions and in some rural areas where livestock farming creates a lot of jobs. Livestock management practices are also challenging, particularly in considering how farm labour is organized (Hostiou and Dedieu, 2012). Technology and automation do have a direct impact on number of working hours, but their social effects are controversial (Dumont *et al*., 2018a).
∙ Social and cultural factors. This interface incorporates a range of topics such as cultural heritage, ethical considerations (e.g. on animal welfare) and reglementary rules relative to land use (e.g. agri-environmental schemes). The relationships between animal health, consumption of animal products and human health are embodied in the one-health framework (Sabate *et al*., 2016). In the global North, animal welfare is a major social concern, resulting in controversies revolving around industrial livestock systems (Delanoue *et al*., 2018). Livestock systems also contribute to creating readily identifiable cultural landscapes and often support local gastronomy. Social and cultural factors cannot yet be quantified at a large scale using available databases, but some attempts have been initiated locally using qualitative approaches (Beudou *et al*., 2017). Our framework provides for this important interface to be evaluated along the lines of some recent attempts for a more global evaluation of livestock farming systems or landscapes (Plieninger *et al*., 2013; Röös *et al*., 2016).

Five interfaces were defined representing how any livestock farming system interacts with its ecological and socio-economic context. First, we considered two major market types. These are (i) the market for animal products (i.e. provisioning services in Ryschawy *et al*., 2017a) and (ii) the purchasing of inputs required for production (arable land devoted to soya beans and corn for animal feeds, chemical fertilizers, etc.) that are imported to the system production area (Chaudhary and Kastner, 2016). To do this, we considered that input-based systems are integrated in internationally traded food markets and therefore we needed to consider both their local and global environmental impacts (Marsden, 2011). Among the five interfaces, the three that are the most directly impacted by considering the two different types of markets are as follows:

∙ Markets. The market interface accounts for the type (pictograms) and quantity of agricultural products, and for opportunities (quality-labelled products represented by the green outward-pointing arrow in Figure 1) and threats (little added-value for young beef represented by red hatches in the outward-pointing arrow, fluctuating prices of crops represented by the red inward-facing arrow) resulting from marketing agreements along the livestock agri-food chains.
∙ Inputs. While local production of livestock feed (Magne *et al*., 2016; Table 2) is represented by green circular arrows within the barn, the red inward-facing arrow on the inputs interface represents the quantity of exogenous inputs that are required either directly (fertilizers, veterinary products, etc.) or indirectly (pesticides, non-renewable energy for concentrate feeds, etc.) for livestock production. Quantification of these indicators is highly dependent on (i) the allocation of land area to different products and by-products, (ii) categories of inputs and (iii) assumptions made to quantify their relative importance (e.g. blue, green and grey water).
∙ Environment and climate. The interface of the livestock systems with its biophysical environment accounts for the different environmental compartments, that is, different pictograms are used for air, water and soil quality, and biodiversity (Millenium Ecosystem Assessment (MEA), 2005; FAO, 2006). The relative importance of positive and negative impacts of livestock farming on the environment is represented by the size and colour of the outward pointing arrow. In Figure 1, high levels of carbon storage, water quality and biodiversity associated to permanent grasslands are represented by the large green outward-pointing arrow, while high levels of CH_4_/kg of product led to hatching red this arrow. Zhang *et al*. (2007) and Duru *et al*. (2015) have also quantified that agricultural production is also the result of biological regulations, soil fertility and erosion control provided by grassland ecosystems to farmers. So-called ‘input services’ are represented by a green inward-facing arrow in Figure 1. On this interface, we account for the impact of livestock on climate (FAO, 2006) but also for the sensitivity of the livestock systems to climate change (Magne *et al*., 2016), which can lead to hatching red the input services arrow (Figure 2b).

### Coloured arrows represent the impacts and services provided by livestock farming

Thanks to arrow size, the ‘barn’ allows end-users to visualize the relative importance of variables represented along the five interfaces. It should be noted that arrow size can either account for monetary data from traded volumes (on *Markets* and *Inputs* interfaces), number of workers (*Work and employment*) or for the magnitude of effects (*Environment and climate*; *Social and cultural factors*). Outward-pointing arrows have up to three different sizes reflecting the magnitude of the effect. A thin, intermediate and large arrow corresponds to a low, intermediate and large impact/service, respectively. The nature of the interaction is given by arrow colours. Using hatched arrows provide our graphical representation with more flexibility. For instance, there could be a high number of jobs in the livestock agro-food industry (represented by a large green arrow) but low wages in the sector, which leads to hatching this arrow red (see other trade-offs in previous *Markets* and *Environment and climate* sub-sections). Inward-facing arrows represent either opportunities (direct sales on *Markets* interface or input services on *Environment and climate* interface) or external pressures (fluctuating prices on the global market, dependence to external inputs and predation risks, on the *Markets*, *Inputs* and *Environment and climate* interfaces, respectively) that affect livestock production systems. Inward facing arrows can also have three different sizes.

### How to analyse and process information to produce the graphical outcome?

When developing the ’barn’, we used a combination of quantitative and qualitative indicators but focus on quantitative indicators when available. The *Markets* and *Inputs* interfaces could be evaluated through monetary data, which is available through large databases such as Farming Accountancy Data Network or through farmers’ interviews for barns produced at a finer level. The *Work and employment* interface can utilize data on the number of workers in the livestock sector in France from the National Institute for Statistics and Economic studies and from the Agricultural Mutual Assistance Association at NUTS3 level; however, statistical confidentiality may apply at finer level (Ryschawy *et al*., 2017a). Values for the *Environment and climate* interface were standardized between 0 and 1 by Ryschawy *et al*. (2017a). They can then be compared and identified as services or impacts depending on whether the value was higher or below a threshold value of 0.5. For instance, the proportion of counties outside nitrate vulnerable zones (i.e. below the legal threshold of 50 mg/l; Dourmad *et al*., 2017) was used as a water quality indicator. It was considered to be negative when it was below 0.5 and led to a red arrow and a grey water-quality pictogram in Catalonia (Dumont *et al*., 2018b) and Brittany (Dourmad *et al*., 2017; Figure 2). However, when it was greater than 0.5, it was considered as a service and this information was used to develop the green arrow and a blue water-quality pictogram in Franche Comté and Provence (Vollet *et al*., 2017; Dumont *et al*., 2018b). Synthesizing the information on the different environmental compartments into a single outward-pointing arrow was expert-based since there were no common metrics for air, water and soil quality, and biodiversity (Figure 1). This approach was considered valid because experts from various domains and knowing the different livestock production areas were consulted. In addition, the environmental criteria was understood and well accepted, and the number of criteria was small enough to be considered as a group by the experts (Bouyssou, 1990). A literature search revealed very few quantitative indicators for the *Social and cultural factors* interface (Plieninger *et al*., 2013, Beudou *et al*., 2017). Therefore, this interface was analysed using expert knowledge or through knowledge exchange with local stakeholders or policy-makers as suggested by Bammer (2005).

The ‘barn’ aims to explicitly reveal the synergies and trade-offs between services and impacts. Using a number of distinct pictograms allows data to be interpreted on one interface, and limits the loss of information which can occur through aggregation. As Ryschawy *et al*. (2017a) suggested, no weighing is suggested *a priori* but local stakeholders could prioritize some interfaces according to their viewpoints on livestock systems. The absence of a pictogram or of an arrow may not mean that the factor was absent, but rather that it was considered negligible compared with other livestock production areas or farming systems. Pictograms are not limited in number, since new pictograms could be added if needed for other livestock production areas (Dernat *et al*., 2019).

## Application of the barn to other contexts

In the previous section, we analysed processing information to produce the graphical representation of the Tarn-Aveyron Basin. In this section, we will use the barn to compare (i) two contrasting livestock production areas (high livestock density *v.* grassland-based), and (ii) the dominant *v.* a niche system within the Tarn-Aveyron Basin.

### Comparing a high livestock density *v*. a grassland-based production area

One of the strengths of the ‘barn’ is the ability to compare livestock production areas by standardizing the indicators through three possible arrow sizes and different types, sizes and colours of pictograms. Figure 2 shows a cross-comparison of the synergies and trade-offs between services and impacts in two contrasting French regions, Brittany and Massif central. Brittany (Dourmad *et al*. 2017) is an example of an area with high livestock densities that has trade-offs between high production and employment on one side and high local environmental impacts on the other that can lead to an overall negative image of livestock production systems by society. In comparison, in the French Massif central, located mainly in the Auvergne region, Protected Designation of Origin (PDO) production (Duru *et al*., 2017; Vollet *et al*., 2017), contributes to grassland-based landscapes with moderate livestock density, improved synergies between production, environmental and cultural services, and a better societal acceptability of livestock production.

The comparison in Figure 2 illustrates how the ‘barns’ can rely on numerical values for services and impacts. The size of the pictograms and arrows match technical and socioeconomic references from DGAGRI – RICA EU 2015 (Hercule *et al*. 2017). In 2015, Brittany had a total of 20.080 livestock farms (46% dairy cattle, 27% pigs and poultry, 9% beef cattle) and Auvergne had 12.100 livestock farms (46% beef cattle, 34% dairy cattle, 8% small ruminants), which corresponds to the animal pictogram size in the two barns (Figure 2). Regional differences in on-farm job numbers (40.360 *v*. 25.290 in Brittany and Auvergne, respectively) and in agricultural production (129.900 *v*. 68.300 euros per farm worker) are also consistent with contrasting arrow size on the *Work and employment*, and *Markets* interfaces. Ruminant production in Auvergne relies more on subsidies than in Brittany (Hercule *et al*., 2017), and so agri-environmental schemes were indicated on the *Social and cultural factors* interface of the Massif central barn. Conversely, in high livestock density areas, policies such as the EU Nitrates Directive aim to regulate the local water and air pollution from intensive farming, and are mentioned on the *Social and cultural factors* interface. Finally, using LCA, it would be possible to quantify off-farm impacts due to feed imports while other variables (biodiversity, water quality, etc.) can be quantified by using longitudinal surveys or networks to determine the size of pictograms and arrows on the *Environment and climate* and *Inputs* interfaces.

### Comparing alternative farming systems in the same region

The ‘barn’ concept could be applied in a same region but at different organizational scales (county, collective of farms, etc.). Doing this may reveal production market-niches, which are often hidden by the dominant socio-technical regime. In the Tarn-Aveyron Basin, local stakeholders have highlighted the reputation and diversity of quality-labelled products associated with various ruminant farming systems, and also with monogastric farms using local maize (geese and fattened ducks) (Moraine *et al*., 2016). The coexistence of these systems provides significant benefits for the landscape and local gastronomy and gives tourists a high environmental quality experience. However, the degree of integration between crop and livestock farms may be variable and limited, and there are conflicts over water use because of the high quantities of water required to produce maize silage (Murgue *et al*., 2015).

**Figure 3:**
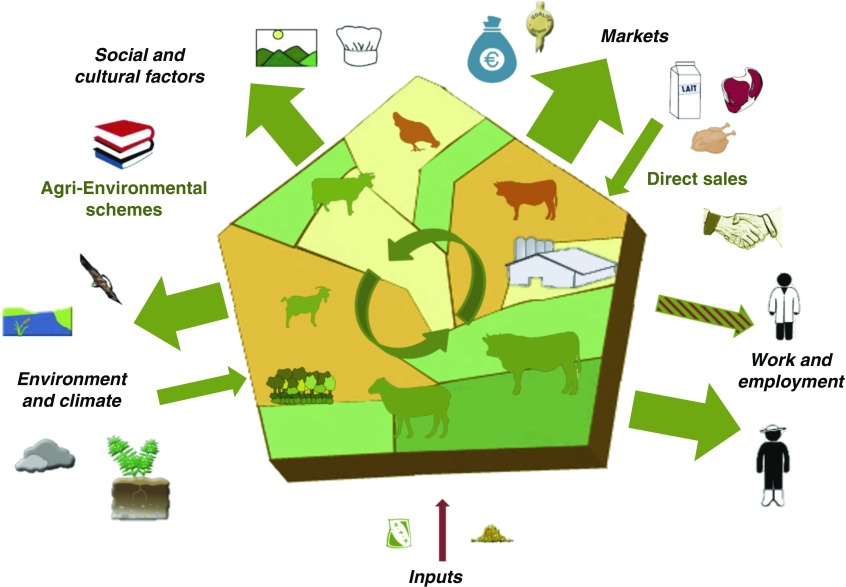
Application of the ‘barn’ framework to a niche-system in the Tarn-Aveyron Basin. It is composed of a collective of seven organic farmers that are developing resource exchanges between neighbours to become self-sufficient in fertilizer and animal-feed inputs (Ryschawy *et al*., 2019). The internal exchanges helps reduce external inputs, increase soil carbon storage and resulted in a more balanced bundle of services with more green arrows on the different interfaces. See Figure 1 for pictogram meaning.

Applying the multi-level perspectives framework of Schot and Geels (2007) to this crop–livestock region, it could be considered that systems with low level of technical integration between crops and livestock are the dominant socio-technical regime for crop–livestock farming (Figure 1), while highly integrated crop–livestock system maximizing interactions between animals, crops and grasslands are a market niche system. In Figure 3, the ‘barn’ framework was applied to this type of market-niche system within the Tarn-Aveyron Basin. The ‘barn’ in this example, was developed using knowledge exchange between farmers, their adviser and researchers as a tool (Bammer, 2005). The market-niche is comprised of seven specialized farms (three crop farms and four livestock farms) on a study area of 201 ha (Ryschawy *et al*., 2019). The producers on these farms are developing resource exchanges between neighbours to become self-sufficient in fertilizer and animal-feed inputs. Currently, livestock farmers are not totally self-sufficient in producing animal feed; they need to buy four tons of sunflower and 18 tons of maize produced by crop farmers within the collective. In addition, the collective’s self-sufficiency in livestock feed and soil conditions were enhanced since crop farmers produce 44 tons of barley–pea mixtures and 8 tons of alfalfa in their rotations. Livestock farmers provide 100 tons of manure per year to crop farmers which help limit their mineral fertilizer inputs. The size of green circular arrows within the barn was increased to represent nutrient cycling through feed crops and manure exchanges within the collective, while the red inward-facing arrow on the *Inputs* interface was reduced.

There were still logistical and social issues because of the increased workload and the need for a different set of management skills. However, the trade-offs between individual and collective benefits were acceptable and resulted in better working conditions and greater self-sufficiency in decision-making at the collective level (Moraine *et al*., 2016). The group in the study was organic and direct markets its products. The collective’s *Market* interface was thus clearly improved (Figure 3) compared to the common practice of selling young live cattle to the Italian market, where returns are poorer since the animals are not fattened (Figure 1). There were fewer people employed in the supply-chain sector (Figure 3) because the farmers in the collective focus on reducing off-farm inputs resulting in lower employment in input-suppliers and commercialization firms. Finally, the *Social and cultural factors* interface benefited from reduced water use linked to the reduced production of maize silage, and from the positive image provided by quality-labelled products in a preserved landscape mosaic.

## Strengths and weaknesses of the ‘barn’

The main strength of the ‘barn’ is the possibility to integrate multi-disciplinary knowledge, especially with stakeholders, and therefore account for generic and local knowledge. Multicriteria analysis and methods examining ‘bundles of services’ highlight the synergies and trade-offs between services but most assessments aggregate indicators (Table 1), which suggests that negative effects on one dimension could be counterbalanced by an improvement on another. This, however, requires caution due to the complex nature of these interactions, and because different stakeholders can develop their own system of value for the impacts and services provided by livestock farming (Oteros-Rozas *et al*., 2012; Clark *et al*., 2017). Reaching high performance in all dimensions of sustainability is difficult to achieve, as shown by the frequent trade-off between livestock production on one side and most regulating and cultural services on the other (Turner *et al*., 2014; Dumont *et al*., 2018b). A primary innovation of the ‘barn’ is that it explicitly represents all the impacts of livestock farming. For example, we utilized a red arrow and a grey water pictogram to explicitly visualize water pollution in Brittany and Catalonia (Dourmad *et al*., 2017; Dumont *et al*., 2018b) in contrast to the bundle of services method that accounts only for services (Turner *et al*., 2014; Ryschawy *et al*., 2017a). Therefore, no hidden cost or benefit of livestock farming is excluded from the graphical representation, and local know-how can be used to fill gaps in scientific knowledge when no validated indicators are available (Dolman *et al*., 2014). Win-win solutions are more likely to emerge when they result from collective decisions that include the point of view of various stakeholders (Howe *et al*., 2014). This highlights the importance of accounting for the whole socio-ecological system as was done with the ‘barn’.

The ‘barn’ graphically summarizes the ecological and socio-economic aspects of livestock farming. It has already been used as a pedagogical approach with advanced students to compare the synergies and trade-offs between impacts and services across a range of European livestock production areas facing highly contrasted pedoclimatic and livestock density conditions. This appeared as a powerful tool that made them realize there is no perfect situation since all livestock regions have advantages and limitations, but in some case studies more balanced bundles of services were provided (Ryschawy *et al*., 2017b; Vollet *et al*., 2017; Dumont *et al*., 2018b; Figure 3). Such a graphical tool could be easily adapted to other agricultural systems, while adding some pictograms and using the same way of quantifying the indicators based on available quantitative indicators and/or expert-knowledge. So far, a total 24 barns have been built from case studies in France, Ireland, Spain, Switzerland and Germany but the tool has not been tested yet outside Europe. It is noteworthy that analysing and processing information to quantify arrow and pictogram sizes more easily applies when comparable data are available for the different livestock production areas or systems being compared.

The ‘barn’ is a structured graphical tool that can help a large diversity of stakeholders (farmers, individuals involved in supply chains, policy-makers, Non-governmental organizations, etc.) understand key opportunities and threats related to livestock production, and quickly identify relationships between the main features of a system at a given scale. As proposed by Bammer (2005), such a framework could be a way of implementing science through knowledge exchange with local stakeholders. Any stakeholder can stress the relevant challenges for local livestock farming and share his views on any of the synergies, trade-offs, co-ordinations or scenarios that are visualized using the ‘barn’. Decisions and prioritization between scenarios can then be discussed based on expert opinion, as shown by Lamarque *et al*. (2011) for Alpine grassland-based landscapes and Moraine *et al*. (2016) for a crop–livestock region. In the Fourme de Montbrison PDO area, the ‘barn’ was used for the first time as serious-game in October 2018 on a large stakeholder panel (*n*=98) from this territory (Dernat *et al*., 2019). It facilitated the PDO actors’ recognition of their natural, technical, economic and institutional environment. It also helped establish a collaboration between stakeholders that provides for their individual representations and leads to a more global and unified view of their territory (Dernat *et al*., 2019). The objective of building alternative scenarios for the future of this PDO production area was achieved and this exercise proves the operationality of the ‘barn’.

As with any visual representation, the heuristic scope of the ‘barn’, however, remains limited as not all interactions can be described. The ‘barn’ focusses on interactions related to livestock systems. We have chosen a spatial structure, with reference to land use that can be either a farm, a farming system or a production area. Skills and inter-knowledge networks cannot be simply visualized using such a diagram, even though they contribute to system dynamics (Dumont *et al*., 2018a). Not all interlocking territories can also be represented in a single diagram, but it is noteworthy that exported impacts embodied in international food trade are explicitly represented on the *Inputs* interface, while other global impacts are accounted for on *Markets* and *Environment and climate* interfaces. Our approach could be extended to or complementary to food systems framework as suggested by Sabate *et al*. (2016), in which the focus is more on consumers and health issues.

The ‘barn’ has been presented here as a static representation of livestock production areas. Irreversibilities and non-linearities also are not explicitly represented but this is also true in all current types of graphical representation. Still, the ‘barn’ can be used to represent the trajectory of change of any livestock farming system by considering the system representation at current time and either its historical perspective or projected representation in the future according to various evolutionary scenarios. Trajectories of French livestock regions have been studied by Domingues *et al*. (2018) based on national databases from 1938 to 2010. This type of data could thus be used to develop a temporal analysis of the trajectories of change of French livestock regions and the underlying drivers. When using the barn in the Fourme de Montbrison PDO area, two evolutionary scenarios were represented. They were, a ‘business as usual’ scenario and an ‘enforced territorial organization’ scenario which aimed to create more value by better organizing the local transformation chain and enhancing the local resource use (Vollet *et al*., 2017). The ‘barn’ could be a first step to identify the opportunities and threats to the future of livestock production from the farm up to the regional level. Its operationality can help identify transition pathways for improving livestock sustainability in the future as recommended by Pigford *et al*. (2018).
